# Upregulation of dihydropyrimidinase-like 3 (DPYSL3) protein predicts poor prognosis in urothelial carcinoma

**DOI:** 10.1186/s12885-023-11090-z

**Published:** 2023-06-28

**Authors:** Peir-In Liang, Hong-Yue Lai, Ti-Chun Chan, Wei-Ming Li, Chung-Hsi Hsing, Steven K. Huang, Kun-Lin Hsieh, Wen-Hsin Tseng, Tzu-Ju Chen, Wan-Shan Li, Huan-Da Chen, Yu-Hsuan Kuo, Chien-Feng Li

**Affiliations:** 1 Department of Pathology, Kaohsiung Medical University Hospital, Kaohsiung Medical University, Kaohsiung, 807378 Taiwan; 2 Department of Medical Research, Chi Mei Medical Center, Tainan, 710402 Taiwan; 3 National Institute of Cancer Research, National Health Research Institutes, Tainan, 704016 Taiwan; 4 Department of Urology, Kaohsiung Medical University Hospital, Kaohsiung Medical University, Kaohsiung, 807378 Taiwan; 5 Department of Urology, School of Medicine, College of Medicine, Kaohsiung Medical University, Kaohsiung, 807378 Taiwan; 6 Center for Liquid Biopsy and Cohort Research, Kaohsiung Medical University, Kaohsiung, 807378 Taiwan; 7 Department of Urology, Ministry of Health and Welfare Pingtung Hospital, Pingtung, 90054 Taiwan; 8 Department of Anesthesiology, Chi Mei Medical Center, Tainan, 710402 Taiwan; 9 Department of Surgery, Division of Urology, Chi Mei Medical Center, Tainan, 710402 Taiwan; 10 Department of Medical Science Industries, College of Health Sciences, Chang Jung Christian University, Tainan, 711301 Taiwan; 11 Department of Clinical Pathology, Chi Mei Medical Center, Tainan, 710402 Taiwan; 12 Department of Medical Technology, Chung Hwa University of Medical Technology, Tainan, 71703 Taiwan; 13 Department of Pathology, Chi Mei Medical Center, Tainan, 710402 Taiwan; 14 Department of Internal Medicine, Division of Hematology and Oncology, Chi-Mei Medical Center, Tainan, 710402 Taiwan; 15 College of Pharmacy and Science, Chia Nan University, Tainan, 71710 Taiwan

**Keywords:** Dihydropyrimidinase-like 3 (DPYSL3), Upper urinary tracts urothelial carcinoma (UTUC), Urinary bladder urothelial carcinoma (UBUC), Cytoskeleton modification, Mammalian target of rapamycin (mTOR), Ribosomal protein S6 (RPS6), Cellular Myelocytomatosis (C-Myc), Glucose transporter 1 (GLUT1), Metabolic process reprogramming

## Abstract

**Background:**

Dihydropyrimidinase-like 3 (DPYSL3) is a cytosolic phosphoprotein expressed in the nervous system and is crucial for neurogenesis. A previous study showed that increased DPYSL3 expression promotes tumour aggressiveness in pancreatic ductal adenocarcinoma, gastric cancer, and colon cancer. However, the role of DPYSL3 in affecting the biological behaviour of urothelial carcinoma (UC) is not yet understood.

**Methods:**

A UC transcriptomic dataset from the Gene Expression Omnibus and the Urothelial Bladder Cancer (BLCA) dataset from The Cancer Genome Atlas were used for the in silico study. We collected 340 upper urinary tract urothelial carcinoma (UTUC) and 295 urinary bladder urothelial carcinoma (UBUC) samples for the immunohistochemical study. Fresh tumour tissue from 50 patients was used to examine the DPYSL3 mRNA level. In addition, urothelial cell lines with and without *DPYSL3* knockdown were used for the functional study.

**Results:**

The in silico study revealed that DPYSL3 correlated with advanced tumour stage and metastasis development while functioning primarily in the nucleobase-containing compound metabolic process (GO:0006139). *DPYSL3* mRNA expression is significantly upregulated in advanced UC. Furthermore, overexpression of the DPYSL3 protein is significantly associated with the aggressive behaviour of UTUC and UBUC. DPYSL3 expression independently predicts disease-specific survival (DSS) and metastatic-free survival (MFS) in patients with UC. In non-muscle-invasive UBUC, DPYSL3 expression predicts local recurrence-free survival. UC cell lines with *DPYSL3* knockdown exhibited decreased proliferation, migration, invasion, and human umbilical vein endothelial cells (HUVECs) tube formation but increased apoptosis and G1 arrest. Gene ontology enrichment analysis revealed that the enriched processes related to DPYSL3 overexpression in UC were tissue morphogenesis, cell mesenchyme migration, smooth muscle regulation, metabolic processes, and RNA processing. In vivo study revealed *DPYSL3* knockdown in UC tumours significantly suppressed the growth of tumours and decreased MYC and GLUT1 protein expression.

**Conclusions:**

DPYSL3 promotes the aggressiveness of UC cells by changing their biological behaviours and is likely associated with cytoskeletal and metabolic process modifications. Furthermore, DPYSL3 protein overexpression in UC was associated with aggressive clinicopathological characteristics and independently predicted poor clinical outcomes. Therefore, DPYSL3 can be used as a novel therapeutic target for UC.

**Supplementary Information:**

The online version contains supplementary material available at 10.1186/s12885-023-11090-z.

## Background

Urothelial carcinoma (UC) is a common tumour that involves the upper urinary tract (renal pelvis and ureter, UTUC) or urinary bladder (UBUC). UTUC is less frequently seen than UBUC, accounting for 5% ~ 10% of all UC cases worldwide. However, a higher incidence of UTUC is seen in populations associated with Balkan endemic nephropathy, Chinese herb nephropathy, and phenacetin abuse [1–3]. The prevalence of UTUC in Taiwan has progressively increased in recent years, accounting for more than 40% of UC cases. Exposure to aristolochic acid-containing herbal medicines plays a vital role in the high incidence of UTUC in Taiwan [4].

UC has heterogeneous biological behaviours with a wide range of recurrence and progression probabilities. Most patients (~ 75%) with newly diagnosed UBUC have non-muscle invasive bladder cancer (NMIBC). The five-year tumour-specific survival rates for early and advanced UBUC are > 90% and ~ 60%, respectively [5]. In contrast, UTUC patients have a more dismal outcome. Most UTUC patients have advanced tumour stage (> pT2) at the initial diagnosis [6, 7]. The 5-year disease-specific survival rate of patients with advanced disease is less than 50% [8]. Whether UBUC and UTUC harbour the same molecular alterations is controversial. However, some studies have shown that the gene expression profile and the frequently mutated genes of UTUC and UBUC showed some similarities [2, 9]. These findings indicated that some aspects of UBUC and UTUC tumorigenesis might have common pathways. A comprehensive understanding of UC biology is essential for developing new treatment modalities.

Rapid growth and proliferation are characteristics of cancer cells, and require the facilitation of DNA and RNA biosynthesis in tumour cells. Therefore, cancer cells reprogram the nucleotide production and degradation processes to preserve the nucleotide pools to achieve this requirement. Interfering with nucleotide metabolism is a frequently used strategy in cancer treatment [10]. Using the transcriptomic dataset GSE31684 from the Gene Expression Omnibus (GEO) and focusing on the nucleobase-containing compound metabolic process (GO:0006139), we identified DPYSL3 as the gene that is significantly correlated with UBUC patients prognosis.

Dihydropyrimidinase-like 3 (DPYSL3), also called collapsing response mediator protein 4 (CRMP4), is a cytosolic phosphoprotein highly expressed in the nervous system [11–14]. The protein product of the *DPYSL3* gene, located on chromosome 5q32, is crucial for many aspects of neurogenesis, including neuronal differentiation, neurite outgrowth, and axonal guidance [14–16]. Overexpression of DPYSL3 in neurons induces filopodia formation and neurite branching [17]. These phenomena are likely a result of DPYSL3-mediated modifications of the neuronal cytoskeleton, such as bundling with F-actin filaments [18]. Previous studies have shown that DPYSL3 has diverse effects on altering cancer cell aggressiveness [19–24]. Decreased DPYSL3 expression in prostate cancer cells enhances cell invasion and migration abilities [23]. Increasing DPYSL3 expression in advanced neuroblastoma improves clinical outcomes [25]. However, the opposite results were observed in pancreatic ductal carcinoma, gastric cancer, and lung cancer. An in vivo study showed that tumours derived from DPSYL3-silenced colon cancer cells have a lower growth rate [22]. Since DPYSL3 actively regulates cancer cell invasion and proliferation, understanding its role in promoting UC cell aggressiveness is essential.

In this study, we revealed that DPYSL3 overexpression in UC is associated with tumour aggressiveness. Knock down of DPYSL3 expression in UC cell lines decreased tumour invasiveness, suppressed angiogenesis, induced apoptosis, and altered energy metabolic processes. This study showed the molecular function of DPYSL3 in UC tumorigenesis, indicating that DPYSL3 may be used as a therapeutic target in the future.

## Methods

### Data mining of public databases

The GSE31684 dataset (http://www.ncbi.nlm.nih.gov/geo/query/acc.cgi?acc = GSE31684) from the NCBI GEO database (GEO, National Center for Biotechnology Information, Bethesda, MD, USA) was selected for transcriptome expression profile analysis. This dataset contains microarray-based expression profiles of surgical specimens from 93 UBUC patients. The investigation was performed with Nexus Expression 3 software (BioDiscovery, EI Segundo, CA, USA). The significantly differentially expressed transcripts in advanced UBUC (pT2-pT3), compared to early-stage UBUC (pTa-pT1), were identified using supervised comparative analysis. In addition, transcripts related to the “nucleobase-containing compound metabolic process” (GO:0006139) were selected for further analysis. Finally, survival analyses of the patients with high and low transcript expression were conducted to validate the impact of the identified genes on survival.

### Study population

The cohort for the immunohistochemical study consisted of 340 samples of UTUC and 295 samples of UBUC collected at ChiMei Medical Hospital between 1984 and 2004. All subjects were newly diagnosed patients who received curative surgery without prior therapy. All UBUC patients with stage pT3 or higher tumours received radical cystectomy followed by cisplatin-based adjuvant chemotherapy, regardless of the status of nodal metastasis. For UTUC, some patients (*n* = 29) with pT3 or pT4 tumours received additional adjuvant therapy after surgery. The clinical data were collected retrospectively from the patient medical charts. The clinicopathological evaluation criteria were mentioned in a previous study [26]. The pathological slides of all patients were reassessed to collect the pathological data. Another cohort of 20 UBUC patients from the same institution was used for quantitative mRNA analysis. The Institutional Review Board approved this study (IRB10501-005).

### Quantitative real-time PCR

Fresh tumour tissues were collected and preserved by snap freezing. We used the VeritasTM automated laser capture microdissection (LCM) system (Arcturus Engineering, USA) to isolate pure UCUB cells from the snap-frozen samples. Three 7 μm serially sliced sections were placed onto a PEN-membrane slide and stained with a HistoGene LCM Staining Kit. Approximately 1500 UCUB cells were isolated from each fresh sample. The RNA extracted using the RNeasy Mini Kit (QIAGEN) was used for cDNA synthesis. The transcript abundance of *DPYSL3* (Hs00910737_ml) mRNA was measured with *POLR2A* (Hs01108291_m1) mRNA used as an internal control. In addition, the fold change in DYPSL3 expression was calculated. The procedure details are documented in our previous work [27].

### Immunohistochemical study

Sections of formalin-fixed paraffin-embedded tissue with a thickness of 4 μm were prepared according to the standard procedure. The sections were incubated with antibodies (Table S1a) for an hour after antigen retrieval. We used the DAKO ChemMate Envision Kit (K5001; Agilent, USA) to detect the primary antibodies. A tumour cell was considered to exhibit positive staining when the brown signal was found in the cellular compartment corresponding to the localization of the target proteins. For surgical specimens, three 1 mm tissue cores from representative tumour areas were removed from the formalin-fixed paraffin-embedded (FFPE) blocks of each patient and used to construct recipient blocks of tissue microarrays (TMAs).

Two pathologists (PIL & CFL) blinded to the clinical data individually evaluated the stained slides to interpret and score the immunoreactivity of DPYSL3. The percentages of tumour cells expressing different levels of chromogen intensity were obtained. The H-score was calculated using the equation,

$$\mathrm H-\mathrm{score}=\Sigma P_i(i+1),$$

where *Pi* indicates the percentage of tumour cells (0% to 100%) with a specific intensity, and *i* denotes the intensity (0 to 4 +). This equation generates a score ranging from 100 to 500, where 100 indicates that all tumour cells were negative, and 500 indicates that 100% of the tumour cells stained strongly for DPYSL3 [28, 29]. UBUC samples with an H-score higher than the median H-score of all UBUC samples were considered to exhibit high expression and those with a H-score lower than the median H-score of all UBUC samples were considered to exhibit low expression. A similar method was also applied to analyse UTUC samples.

### Cell culture and stable DPYSL3 knockdown clones establishment

The UC cells RT4, T24, BFTC905, and BFTC909 were obtained from the Bioresource Collection and Research Center (Hsinchu, Taiwan). UMUC3 and TCCSUP cells were acquired from the American Type Culture Collection (ATCC, Manassas, VA 20108, USA). The growth conditions and medium for each UC cell line were implemented as recommended. After screening DPYSL3 protein levels using Western blot analysis, two cell lines, BFTC909 and T24, were selected to generate stable clones. The selected cells were infected with lentivirus carrying a short hairpin RNA sequence to knock down *DPYSL3* in UC cells. The procedure details are documented in our previous work [30]. The lentivirus was purchased from the National RNAi Core Facility, Genomic Research Center of the Institute of Molecular Biology, Academia Sinica, Taiwan, and the shRNA sequences were pLKO.1-*shLacZ* (TRCN0000072223: 5′-TGTTCGC ATTATCCGAACCAT-3′), pLKO.1-*shDPYSL3*#1 (TRCN0000046848, 5′-CGGCATAGATGGAACCCATTA-3′), and pLKO.1-*shDPYSL3*#2 (TRCN 0000046850, 5′-GCGGCAGAGTACAACATCTTT-3′).

### Western blot analysis

Total protein (25 μg) extracted from each UC cell line was separated on a 4–12% SDS‒PAGE gel (NuPAGE; Invitrogen, USA) and transferred onto a polyvinylidene fluoride (PVDF) membrane (Amersham Biosciences, UK). The protein containing membrane was then blocked using 5% skim milk in Tris-buffered saline with Tween® 20 (TBST) at room temperature for one hour. Next, the membrane was incubated with antibodies (Table S1b) at 4 °C overnight. An anti-GAPDH antibody (6C5, 1:10,000; Millipore, USA) was used as a loading control antibody. The membranes were then incubated with secondary antibodies at room temperature for 90 min. Proteins were detected by enhanced chemiluminescence reagents (Amersham Biosciences, UK) and protein expression was semiquantitatively measured using densitometry.

### Cell proliferation assay

This experiment was carried out using the Cell Proliferation Assay Kit (Fluorometric; BioVision, USA) and was performed according to the manufacturer’s recommendation. In brief, approximately 1500 cells were plated on a 96-well dish and incubated at 37 ℃ and 5% CO_2_. At the end of the incubation period (24, 48, and 72 h), 25 µl of lysis buffer and nuclear staining mixture was added. After a short incubation period, a microplate reader (GM3000; Promega Corp, USA) was used to measure the fluorescence intensity with the excitation/emission wavelengths set at 480/538 nm. *The procedure details* are documented in our previous work [30].

### Cell migration and invasion assays

The Falcon™ FluoroBlok HTS 24-Well Insert System (351,152; Corning, USA) and the ECMatrix Cell Invasion Assay (ECM554; MilliporeSigma, USA) were used, and the assays were performed as recommended. In brief, 1 × 10^5^ cells suspended in serum-free culture medium were seeded in the upper chambers. DMEM supplemented with 10% FBS (11,960,044; Thermo Fisher Scientific, USA) was added to the lower chambers, and the plates were incubated for six hours at 37 ℃ and 5% CO_2_. Cells migrating to the bottom surface of the insert membranes were dissociated from the membrane and detected by applying CyQUANT GR dye. The percentage of the fluorescence signal in the experimental groups relative to the control groups indicates the cell migration and invasion capabilities.

### Flow cytometric analyses of apoptosis and the cell cycle

For apoptosis assays, approximately 1 × 10^5^ UC cells were incubated with the components of an Annexin V-FITC kit (331200; Thermo Fisher, USA) at room temperature for 15 min and protected from light. For cell cycle assays, UC cells were fixed in 70% ethanol overnight at -20 ℃. Next, the cells were washed and resuspended in propidium iodide (PI)/ribonuclease staining buffer (550825; BD Biosciences, USA) for 15 min in the dark. These samples were then surveyed using a NovocyteTM flow cytometer (ACEA Biosciences, USA). At least three independent experiments were carried out for each experimental group.

### Human umbilical vein endothelial cells (HUVECs) tube formation assay

Fixed numbers (5 × 10^4^ cells) of HUVECs (ATCC, USA) were incubated in the conditioned mediums collected from the *DPYSL3* knockdown and mock UC cells. They were then seeded in a 24-well plate precoated with Matrigel basement membrane matrix (Corning, USA). The experiment was terminated after six hours of incubation, and tube formation was analysed by measuring the tube formation ability on the images captured under high-quality phase-contrast microscopy.

### Xenograft animal models

Stable BFTC909 sh*LacZ* and sh*DPYSL3*#1 cells were mixed with high-concentration Matrigel (354248; Corning Life Sciences, USA) and injected subcutaneously into SCID Beige male mice (purchased from BioLASCO, Taiwan). The tumour size was recorded at the indicated time using an external calliper. The tumour volume was calculated using the following equation: Volume (mm^3^) = (π/6) × width (mm^2^) × length (mm). The mice were euthanized by CO_2_ asphyxiation on Day 21. The excised tumours were fixed in 10% formalin for further analysis. This study was approved by the Institutional Animal Care and Use Committee of Chi-Mei Medical Center (Approval number 109041701).

### Gene ontology (GO) enrichment analysis

The differentially expressed transcripts associated with *DPYSL3* overexpression in UC were identified using the BLCA dataset in TCGA. The analysis was performed online through the c-cBioPortal platform (https://www.cbioportal.org/). The top 500 genes that were positively and negatively coexpressed with DPYSL3 were then subjected to GO enrichment analysis (http://geneontology.org/) to reveal the enriched biological processes.

### Statistical analysis

All analyses were performed using IBM SPSS version 19 (IBM Corp, USA). The correlations between DPYSL3 expression and clinicopathological features were assessed using the chi-square test. The impact of DPYSL3 expression on patient outcomes was evaluated using Kaplan‒Meier survival analysis. We performed a log-rank test to analyse differences in survival. Disease-specific survival (DSS), metastasis-free survival (MFS), and local recurrence-free survival (LRFS) were selected as the clinical endpoints. DSS is the time elapsed from initial surgery to tumour-associated death. MFS is defined as the time from surgery to metastasis. LRFS is defined as the time from transurethral resection of the bladder tumour to tumour recurrence. A Cox proportional hazards regression model was developed to identify independent predictors for patient survival. Patients who were alive without events were censored at the last follow-up. A difference was considered significant if the *p-*value was ≤ 0.05.

## Results

### DPYSL3 overexpression in UBUC was associated with tumour aggressiveness and poor patient survival

**Fig. 1:**
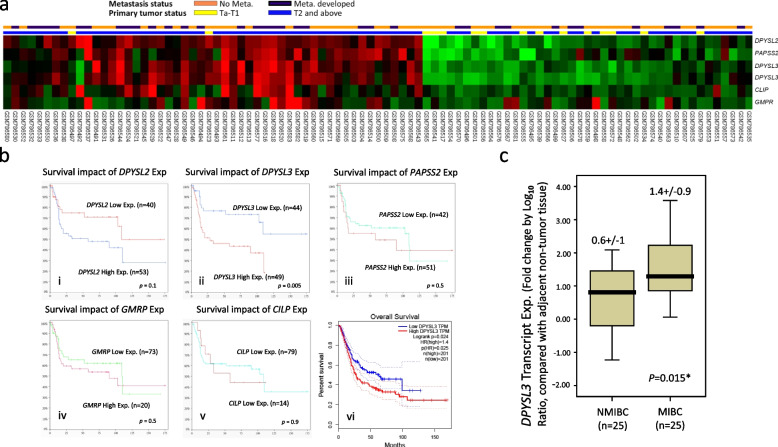
In silico analyses of urinary bladder urothelial carcinoma (UBUC) datasets in the public domain. **a** Using the dataset, GSE32894, in Gene Expression Omnibus with a focus on the nucleobase-containing compound metabolic process (GO:0006139) term, a tumour cluster composed of mostly advanced-stage UBUC was found to have higher expression of five related transcripts (6 probes); **b** Of these transcripts, only (ii) *Dihydropyrimidinase-like 3* **(***DPYSL3)* is significantly associated with survival (*p* = 0.005); (vi) TCGA dataset analysis confirmed our finding; **c** We used 50 UBUC samples to validate this finding and showed higher *DPYSL3* mRNA levels in muscle-invasive bladder cancer than in non-muscle-invasive bladder cancer

The GSE31684 dataset contains data for 93 UBUC patients. Eighty-four percent and thirty percent of these patients have MIBC and lymph node metastasis, respectively. Five transcripts (*DPYSL2, PAPSS2, DPYSL3, CILP,* and *GMPR*) associated with nucleobase-containing compound metabolic process (GO:0006139) were correlated with UBUC invasiveness and metastasis (Fig. 1a and Table 1). Among these transcripts, DPYSL3 had the highest log2 fold difference when comparing the MIBC vs. NMIBC groups (2.04-fold) and the distal metastasis events vs. no metastasis groups (0.77-fold). Correlation analysis with patient survival outcomes in the dataset revealed that *DPYSL3* was the only transcript that showed a significant survival impact in these patients (*p* = 0.005, Figs. 1bi-v). We confirm this finding using the BCLA dataset from The Cancer Genome Atlas (TCGA) program. Analysis of the data online using Gene Expression Profiling Interactive Analysis (GEPIA) demonstrated that high DPYSL3 expression predicts worse overall survival (*p* = 0.024, Fig. 1bvi).

**Table 1:** The significantly altered genes belong to the biological process GO:0006139

**Probe**	**MIBC vs. NMIBC**		**Distal Meta. vs. Non-Meta **^**&**^		**Gene Symbol**	**Gene Title**	**Biological Process**
	**Log2 Ratio**	***p*-Value**	**Log2 Ratio**	***p*-Value**			
**200762_at**	**1.06**	0.002*	0.62	0.016*	*DPYSL2*	dihydropyrimidinase-like 2	cell differentiation, multicellular organismal development, nervous system development, nucleobase; nucleoside; nucleotide and nucleic acid metabolic process, signal transduction
201430_s_at	1.21	< 0.001*	0.34	0.015*	***DPYSL3***	dihydropyrimidinase-like 3	nervous system development, nucleobase; nucleoside; nucleotide and nucleic acid metabolic process, signal transduction
201431_s_at	2.04	< 0.001*	0.77	0.002*	***DPYSL3***	dihydropyrimidinase-like 3	nervous system development, nucleobase; nucleoside; nucleotide and nucleic acid metabolic process, signal transduction
203060_s_at	1.34	< 0.001*	0.58	0.037*	*PAPSS2*	3’-phosphoadenosine 5’-phosphosulfate synthase 2	nucleobase; nucleoside; nucleotide and nucleic acid metabolic process, skeletal development, sulfate assimilation
204187_at	0.15	0.045*	0.17	0.003*	*GMPR*	guanosine monophosphate reductase	metabolic process, nucleobase; nucleoside; nucleotide and nucleic acid metabolic process, nucleotide metabolic process, response to cold
206227_at	0.66	0.002*	0.54	< 0.001*	*CILP*	cartilage intermediate layer protein; nucleotide pyrophosphohydrolase	nucleobase; nucleoside; nucleotide and nucleic acid metabolic process

*MIBC* muscle-invasive bladder cancer, *NMIBC* non-muscle invasive bladder cancer, *Meta* metastasis

^*^Statistically significant

^&^Development of subsequent metastasis

To confirm the in silico analysis results, we evaluated the relationship between DPYSL3 mRNA expression levels in UBUC and tumour aggressiveness. The results showed that advanced-stage UBUC (pT2-pT4) had significantly higher DPYSL3 mRNA expression than early-stage UBUC (pTa-pT1) (*p* < 0.001, Fig. 1c). This result suggests that DPYSL3 plays a significant role in urothelial carcinogenesis.

### DPYSL3 protein overexpression is associated with aggressive clinicopathological features and results in poor survival outcomes in UBUC and UTUC

**Fig. 2:**
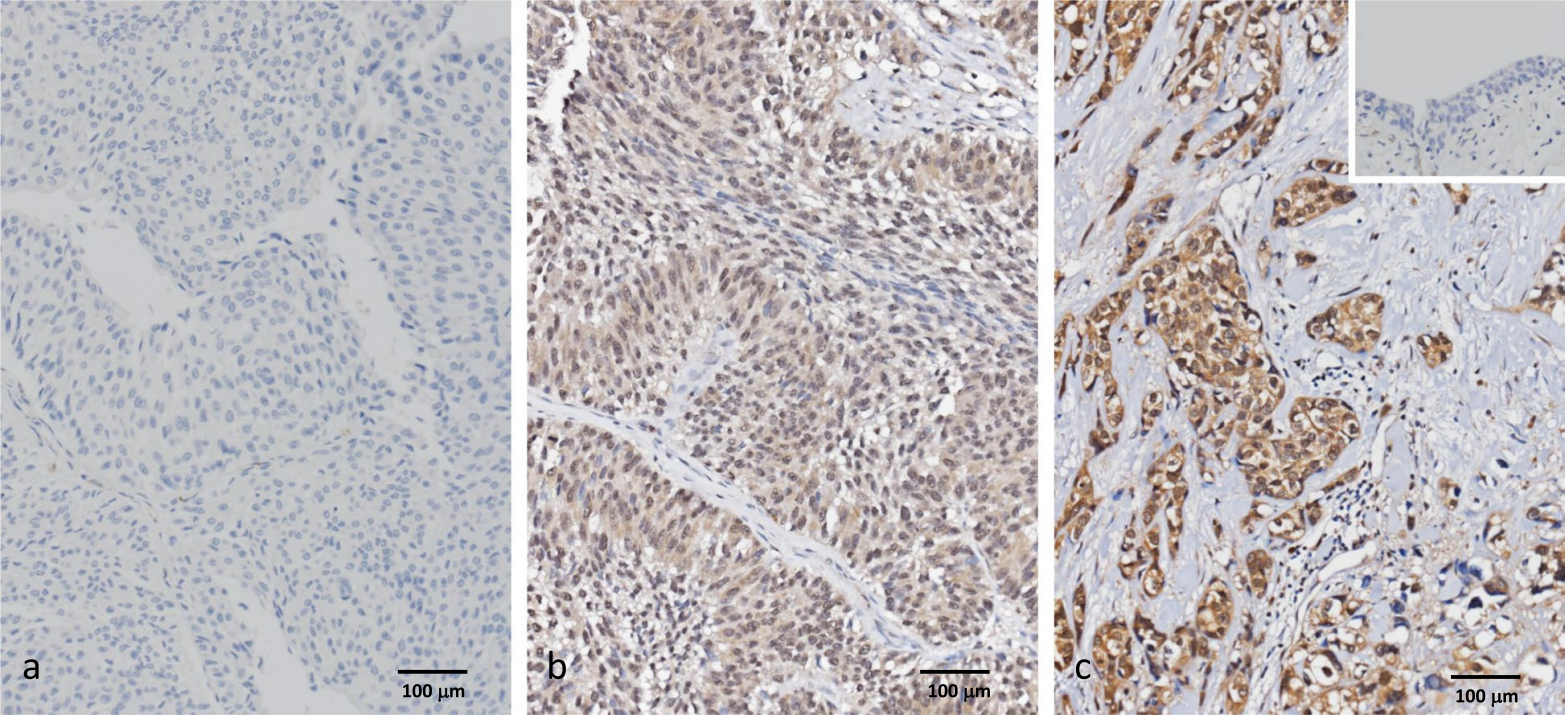
Dihydropyrimidinase-like 3 (DPYSL3) immunohistochemical staining in various stages of urothelial carcinoma. **a** A noninvasive papillary urothelial carcinoma (pTa) is negative for DPYSL3; **b** an early invasive urothelial carcinoma (pT1) shows a low to moderate degree of DPYSL3 expression; **c** a muscle-invasive bladder carcinoma (> pT2, MIBC) shows diffuse, strong DPYSL3 expression. (Magnification, 200 ×)

The DPYSL3 protein shows variable expression in UBUC and UTUC (Fig. 2 and Table 2). In UTUC, high DPYSL3 expression was associated with advanced-stage (*p* < 0.001), lymph node metastasis (*p* < 0.001), high histological grade (*p* < 0.001), vascular invasion (*p* < 0.001), perineural invasion (*p* < 0.001), and a high mitosis rate (*p* < 0.001). In patients with UBUC, increased expression of DPYSL3 was frequently related to muscle-invasive bladder cancer (MIBC) (*p* < 0.001), lymph node metastasis (*p* = 0.012), vascular invasion (*p* < 0.001), and perineural invasion (*p* = 0.021). These observations indicate the importance of DPYSL3 in promoting UC aggressiveness.

**Table 2:** Correlations between dihydropyrimidinase-like 3 (DPYSL3) expression and other important clinicopathological parameters in urothelial carcinomas

**Parameter**	**Category**	**Upper Urinary Tract Urothelial Carcinoma**				**Urinary Bladder Urothelial Carcinoma**			
		**Case No**	**DPYSL3 Expression**		***p*-Value**	**Case No**	**DPYSL3 Expression**		***p*-Value**
			**Low**	**High**			**Low**	**High**	
Gender	Male	158	75	83	0.4	216	106	110	0.7
	Female	182	95	87		79	41	38	
Age (years)	< 65	138	70	68	0.8	121	55	66	0.2
	≥ 65	202	100	102		174	92	82	
Tumour location	Renal pelvis	141	66	75	0.2	-	-	-	-
	Ureter	150	83	67		-	-	-	-
	Renal pelvis & ureter	49	21	28		-	-	-	-
Multifocality	Single	278	143	135	0.3	-	-	-	-
	Multifocal	62	27	35		-	-	-	-
Primary tumour (T)	Ta	89	71	18	< 0.001*	84	54	30	< 0.001*
	T1	92	54	38		88	47	41	
	T2–T4	159	45	114		123	46	77	
Nodal metastasis	Negative (N0)	312	168	144	< 0.001*	266	139	127	0.012*
	Positive (N1–N2)	28	2	26		29	8	21	
Histological grade	Low grade	56	44	12	< 0.001*	56	33	23	0.1
	High grade	284	126	158		239	114	125	
Vascular invasion	Absent	234	153	81	< 0.001*	246	137	109	< 0.001*
	Present	106	17	89		49	10	39	
Perineural invasion	Absent	321	169	152	< 0.001*	275	142	133	0.021*
	Present	19	1	18		20	5	15	
Mitotic rate (per 10 high power fields)	< 10	173	110	63	< 0.001*	139	73	66	0.4
	> = 10	167	60	107		156	74	82	

^*^Statistically significant; *RR* relative risk

The univariable and multivariable analysis results of the clinicopathological features are listed in Tables 3, 4 and 5. For UTUC patients (median (interquartile range (IQR)) follow-up time = 38.2 (19.8, 65.7) months), nodal metastasis and perineural invasion are important clinicopathological factors independently correlated with DSS and MFS. In addition, the histological grade is an independent prognostic factor for DSS and multifocality for MFS. For UBUC patients (median (IQR) follow-up time = 23.4 (9.9, 43.2) months), the primary tumour stage (pT) and mitotic rate predict DSS and MFS of patients. Although univariable analysis showed that pT (*p* = 0.019) and histological grade (*p* = 0.01) are correlated with LRFS in NMIBC patients after transurethral resection of bladder tumours (TURBT), they are not independent prognostic factors (Table 5).

**Fig. 3:**
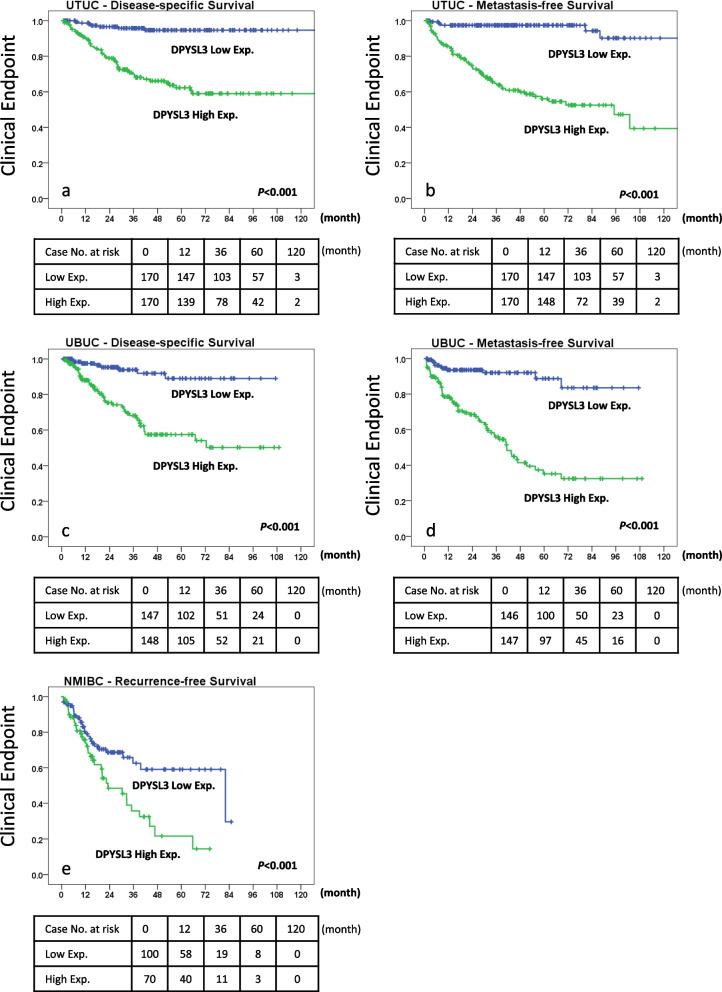
Kaplan‒Meier analyses of survival based on Dihydropyrimidinase-like 3 (DPYSL3) expression in urothelial carcinoma. The plots show that DPYSL3 overexpression is correlated with inferior (**a**) disease-specific survival (DSS) and (**b**) metastasis-free survival (MFS) outcomes in patients with upper urinary tract urothelial carcinoma (UTUC); **c**, **d** a similar pattern is also observed in patients with urinary bladder urothelial carcinoma (UBUC); **e** in patients with non-muscle-invasive UBUC, high DPYSL3 expression is correlated with a higher local recurrence-free survival (LRFS) rate

Patients with UTUC expressing higher levels of DPYSL3 had a significantly higher death rate (31.8% vs. 4.1%, *p* < 0.001) and more frequent metastatic events (35.3% vs. 3.5%, *p* < 0.001, Table 3). In addition, DPYSL3 overexpression was an independent prognostic factor for both DSS (*p* = 0.005) and MFS (*p* < 0.001, Table 3 and Figs. 3a and b). DPYSL3 expression also has a similar effect on UBUC. A higher death rate (29.7% vs. 5.4%, *p* < 0.001) and frequent metastasis (43.2% vs. 7.5%, *p* < 0.001) are seen in UBUC with high DPYSL3 expression (Table 4). Besides, high expression of DPYSL3 also independently predicts dismal DSS (*p* < 0.001) and MFS (*p* < 0.001) outcomes in UBUC patients (Table 4 and Figs. 3a-d). For NMIBC patients, DPYSL3 overexpression was significantly correlated with local recurrence (26.8% vs. 1.0%, *p* = 0.005). DPYSL3 also independently predicted the LRFS (*p* = 0.01) in NMIBC patients after TURBT (Table 5 and Fig. 3e).

**Table 3:** Univariable and multivariable analyses for disease-specific and metastasis-free survivals in upper urinary tract urothelial carcinoma

**Parameter**	**Category**	**Case No**	**Disease-Specific Survival**					**Metastasis-Free Survival**				
			**Univariable Analysis**		**Multivariable Analysis**			**Univariable Analysis**		**Multivariable Analysis**		
			**No. of Event**	***p*-Value**	**RR**	**95% CI**	***p*-Value**	**No. of Event**	***p*-Value**	**RR**	**95% CI**	***p*-Value**
**Gender**	Male	158	28	0.8	-	-	-	32	0.8	-	-	-
	Female	182	33		-	-	-	38		-	-	-
**Age (years)**	< 65	138	26	1	-	-	-	30	0.8	-	-	-
	≥ 65	202	35		-	-	-	40		-	-	-
**Tumour side**	Right	177	34	0.7	-	-	-	38	0.3	-	-	-
	Left	154	26		-	-	-	32		-	-	-
	Bilateral	9	1		-	-	-	0		-	-	-
**Tumour location**	Renal pelvis	141	24	0.008*	-	-	-	31	0.1	-	-	-
	Ureter	150	22		-	-	-	25		-	-	-
	Renal pelvis & ureter	49	15		-	-	-	14		-	-	-
**Multifocality**	Single	273	48	0.003*	-	-	-	52	0.013*	1	-	0.007*
	Multifocal	62	18		-	-		18		2.2	1.2, 3.8	
**Primary tumour (T)**	Ta	89	2	< 0.001*	1	-	0.1	4	< 0.001*	1	-	0.3
	T1	92	9		1.7	0.7, 3.8		15		2.5	0.8, 7.9	
	T2–T4	159	50		5.4	1.2, 25		51		2.1	0.7, 6.7	
**Nodal metastasis**	Negative (N0)	312	42	< 0.001*	1	-	< 0.001*	55	< 0.001*	1	-	0.002*
	Positive (N1–N2)	28	19		3.6	2.1, 6.5		15		2.6	1.4, 4.9	
**Histological grade**	Low grade	56	4	0.022*	-	-	**-**	3	0.003*	1	-	0.1
	High grade	284	57		-	-	**-**	67		1.9	0.8, 4.1	
**Vascular invasion**	Absent	234	24	< 0.001*	1	-	0.4	26	< 0.001*	1	-	0.1
	Present	106	37		1.3	0.7, 2.4		44		1.8	0.1, 3.3	
**Perineural invasion**	Absent	321	50	< 0.001*	1	-	0.005*	61	< 0.001*	1	-	0.048*
	Present	19	11		2.6	1.7, 7.4		9		2.1	1, 4.4	
**Mitotic rate (per 10 high power fields)**	< 10	173	27	0.2	-	-		30	0.1	-	-	
	> = 10	167	34		-	-		40		-	-	
**DPYSL3 expression**	Low	170	7	< 0.001*	1	-	0.005*	6	< 0.001*	1	-	< 0.001*
	High	170	54		3.4	1.5, 8.1		64		6.2	2.5, 15	

^*^Statistically significant; *RR* relative risk

**Table 4:** Univariable and multivariable analyses for disease-specific and metastasis-free survivals in urinary bladder urothelial carcinoma

**Parameter**	**Category**	**Case No**	**Disease-Specific Survival**					**Metastasis-Free Survival**				
			**Univariable Analysis**		**Multivariable Analysis**			**Univariable Analysis**		**Multivariable Analysis**		
			**No. of Event**	***p*-Value**	**RR**	**95% CI**	***p*-Value**	**No. of Event**	***p*-Value**	**RR**	**95% CI**	***p*-Value**
**Gender**	Male	216	41	0.4	-	**-**	**-**	60	0.3	-	-	-
	Female	79	11		-	**-**	**-**	16		-	-	-
**Age (years)**	< 65	121	17	0.1	-	**-**	**-**	31	0.7	-	-	-
	≥ 65	174	35		-	**-**	**-**	45		-	-	-
**Primary tumour (T)**	Ta	84	1	< 0.001*	1	-	< 0.001 *	4	< 0.001*	1	-	0.007*
	T1	88	9		4.1	1.9, 9.2		23		4	1.2, 14	
	T2-T4	123	42		32	4.3, 250		49		5.8	1.6, 20	
**Nodal metastasis**	Negative (N0)	266	41	< 0.001*	1	-	0.6	61	< 0.001*	1	-	0.1
	Positive (N1–N2)	29	11		1.2	0.6, 2.4		15		1.7	0.9, 3.2	
**Histological grade**	Low grade	56	2	0.001*	-	-	-	5	< 0.001*	1	-	0.5
	High grade	239	50		-	-	-	71		1.5	0.5, 4.1	
**Vascular invasion**	Absent	246	37	0.002*	1	-	0.1	54	< 0.001*	1	-	0.4
	Present	49	15		0.5	0.3, 1	**-**	22		0.8	0.4, 1.4	
**Perineural invasion**	Absent	275	44	< 0.001*	1	-	0.1	66	< 0.001*	1	-	0.2
	Present	20	8		2.2	1, 5.3		10		1.6	0.7, 3.3	
**Mitotic rate (per 10 high power fields)**	< 10	139	12	< 0.001*	-	-	**-**	23	< 0.001*	1	-	0.041*
	> = 10	156	40		-	-		53		1.7	1, 2.9	
**DPYSL3 expression**	Low	147	8	< 0.001*	1	-	< 0.001*	11	< 0.001*	1	-	< 0.001*
	High	148	44		4	1.8, 8.6		64		5.1	2.7, 9.9	

^*^Statistically significant; *RR* relative risk

**Table 5:** Univariable and multivariable analyses for local recurrence-free survivals in non-muscle-invasive bladder carcinoma

**Parameter**	**Category**	**Case No**	**Local Recurrence-Free Survival**				
			**Univariable Analysis**		**Multivariable Analysis**		
			**No. of Event**	***p*-Value**	**RR**	**95% CI**	***p*-Value**
**Gender**	Male	125	46	0.3	-	**-**	**-**
	Female	47	19		-	**-**	**-**
**Age (years)**	< 65	70	30	0.4	-	**-**	**-**
	≥ 65	102	35		-	**-**	**-**
**Primary tumour (T)**	Ta	84	27	0.019*	1	-	0.8
	T1	88	38		1	0.6, 2	
**Nodal metastasis**	Negative (N0)	172	65	n.a	-	-	-
	Positive (N1–N2)	0	0		-	-	-
**Histological grade**	Low grade	54	15	0.01*	1	-	0.1
	High grade	118	50		2	1, 4.2	
**Vascular invasion**	Absent	171	65	0.7	-	-	-
	Present	1	0		-	-	-
**Perineural invasion**	Absent	169	64	0.5	-	-	-
	Present	3	1		-	-	-
**Mitotic rate (per 10 high power fields)**	< 10	94	35	0.2	-	-	-
	> = 10	78	30		-	-	-
**DPYSL3 expression**	Low	101	29	0.004*	1	-	0.01*
	High	71	36		2	1.2, 3.3	

^*^Statistically significant; *RR* relative risk

### *DPYSL3* mRNA interference reverses UC cell proliferation and aggressiveness

**Fig. 4:**
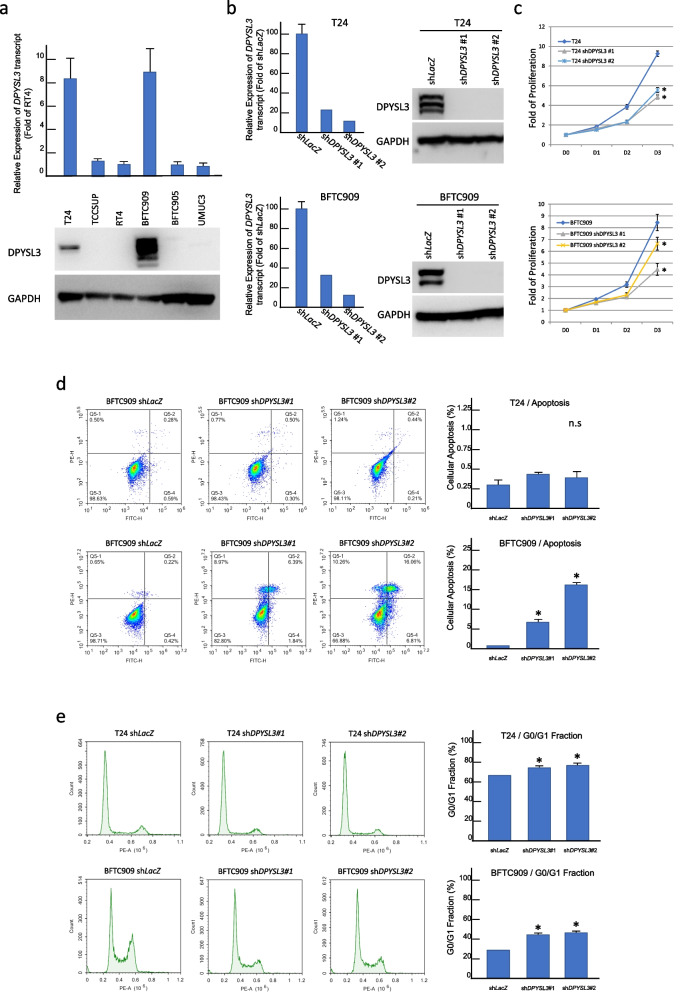
*Dihydropyrimidinase-like 3 (DPYSL3)* knockdown increased urothelial carcinoma (UC) cells apoptosis and altered the cell cycle. **a** T24 and BFTC909 are among the UC cells with increased *DPYSL3* mRNA transcript and high DPYSL3 protein expression; **b** T24 and BFTC909 cells infected with lentivirus carrying *DPYSL3* shRNA exhibited successful knockdown of DPYSL3 expression; **c**  *DPYSL3* knockdown UC cells have a significantly decreased proliferation ability (*p* < 0.05); **d**  *DPYSL3* knockdown T24 cells showed a trend toward an increase in apoptotic activity, and apoptosis was significantly increased in *DPYSL3* knockdown BFTC909 cells (*p* < 0.05); **e**  *DPYSL3* knockdown UC cells exhibited a significantly increased G0/G1 fraction (*p* < 0.05)

**Fig. 5:**
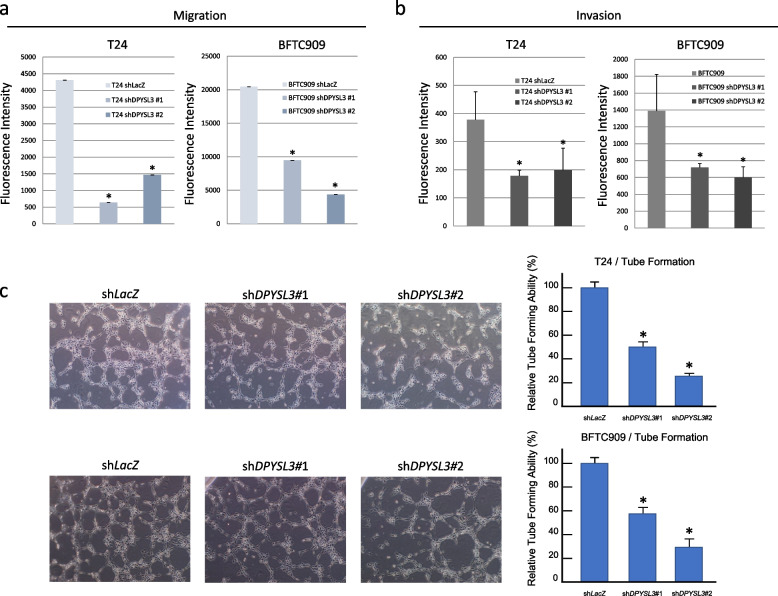
*Dihydropyrimidinase-like 3 (DPYSL3)* knockdown suppressed urothelial carcinoma (UC) cell migration/invasion and hampered angiogenesis. The plots show that UC cells with *DPYSL3* knockdown had a significantly suppressed (**a**) migration ability and (**b**) invasion ability (*p* < 0.05); **c** human umbilical vein endothelial cells (HUVECs) tube formation was significantly hampered after culture in the conditioned medium of *DPYSL3*-knockdown UC cells (*p* < 0.05)

We screened UC cell lines (T24, TCCSUP, RT4, BFTC909, BFTC905, and UMUC3) to understand the expression level of the *DPYSL3* transcript. BFTC909 and T24 have high *DPYSL3* transcript levels among these UC cell lines. Western blots confirmed that the corresponding protein levels were equally elevated (Figs. 4a and S1). To generate stable clones of UC cells with low DPYSL3 expression, we infected BFTC909 and T24 cells with lentivirus carrying sh*DPYSL3* to knock down *DPYSL3* expression (Figs. 4b, S1 and S2). After *DPYSL3* knockdown, UC cells exhibited a significantly decreased proliferation ability (both *p* < 0.05, Fig. 4c). The *DPYSL3* knockdown clones (KD clones) also showed a higher apoptosis rate (BFTC909 *p* < 0.05; T24 not significant, Fig. 4d) and an increased G0/G1 fraction (both *p* < 0.05, Fig. 4e and Table S2). These results suggest that DPYSL3 facilitates UC cell proliferation by positively regulating cell cycle progression and preventing apoptosis. Furthermore, KD clones significantly decreased the migration and invasion ability of BFTC909 and T24 cells (Figs. 5a and b). In addition, the KD clones also exhibited a significantly decreased HUVECs tube formation ability (Fig. 5c). These findings suggested that DPYSL3 enhanced the migration and invasion abilities of UC cells and promoted angiogenesis in UC.

### Genes coexpressed with DPYSL3 in UC are correlated with cell migration, mesenchymal remodelling, and metabolic processes

The 500 transcripts whose levels were most significantly positively or negatively correlated with the DPYSL3 mRNA level in the BLCA dataset of TCGA were extracted and are listed in Tables S3 and S4, respectively. The biological processes involving these genes identified by GO enrichment analysis are displayed in Tables S5 and S6. The top 10 positively associated genes were *MSRB3*, *BNC2*, *CALD1*, *FXYD6*, *FERMT2*, *FGFR1*, *PDLIM3*, *MIR100HG*, *MYLK*, and *TGFB3*. The top 10 biological processes enriched with the positively associated transcripts included “aorta smooth muscle tissue morphogenesis (GO:0060414)”, “mesenchyme migration (GO:0090131)”, “hematopoietic stem cell migration to bone marrow (GO:0097241)”, “elastic fiber assembly (GO:0048251)”, “intramembranous ossification (GO:0001957)”, “relaxation of vascular associated smooth muscle (GO:0060087)”, “direct ossification (GO:0036072)”, “lung growth (GO:0060437)”, “regulation of Rho-dependent protein serine/threonine kinase activity (GO:2000298)”, and “relaxation of smooth muscle (GO:0044557)”. On the other hand, the top 10 genes negatively associated with *DPYSL3* were *PLEKHH1*, *MAP7*, *OVOL1*, *ID1*, *CRB3*, *ESRRA*, *DTX4*, *CHMP4C*, *SSH3*, and *SLC22A5*. The top 10 biological processes enriched with the negatively associated transcripts were “tRNA processing (GO:0008033)”, “regulation of RNA splicing (GO:0043484)”, “fatty acid metabolic process (GO:0006631)”, “mRNA processing (GO:0006397)”, “mRNA metabolic process (GO:0016071)”, “carboxylic acid metabolic process (GO:0019752)”, “oxoacid metabolic process (GO:0043436)”, “organic acid metabolic process (GO:0006082)”, “RNA processing (GO:0006396)”, and “cellular lipid metabolic process (GO:0044255)”.

### DPYSL3 mRNA interference in UC cells suppressed mTOR signalling

**Fig. 6:**
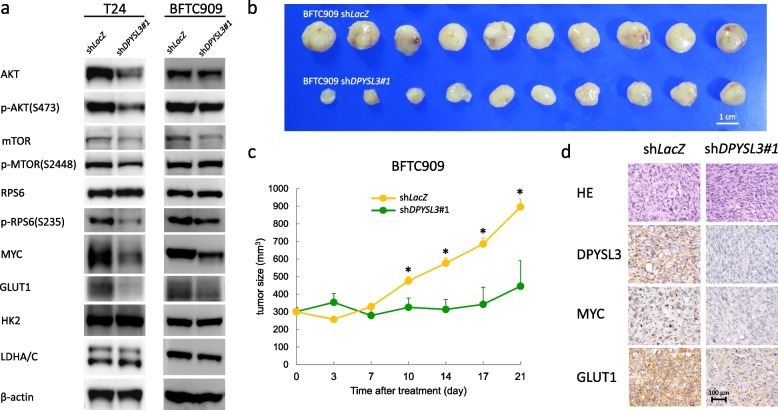
*Dihydropyrimidinase-like 3 (DPYSL3)* knockdown increased phosphorylation of mTORC1 and enhanced UC tumour aggressiveness. The relationship between DPYSL3 and proteins associated with the mTOR pathway was explored by Western blot analysis. **a** p-mTOR, p-RPS6(S235), Myc, and Glut1 were consistently and significantly downregulated in T24 and BFTC909 with *DPYSL3* knockdow. **b** BFTC909-derived xenograft model (*n* = 10 for each group) confirmed the effects of DPYSL3 on promoting tumour growth. **c** The growth rate of tumours derived from sh*DPYSL3*#1 BFTC909 cells was significantly lower than those derived from sh*LacZ* BFTC909 cells in SCID Beige mice. **d** IHC analysis of xenografted samples at day 21 post-inoculation showed that the expression of DPYSL3, MYC, and GLUT1 was suppressed in the DPYSL3 knockdown group, in contrast to the control group (Magnification, 400 ×). Data are shown as the mean ± SEM. Statistical significance: *p < 0.05

The PI3K/AKT/mTOR pathway is essential in regulating energy homeostasis and is frequently affected in UC [31, 32]. Since genes associated with *DPYSL3* were enriched in energy-metabolism processes, we hypothesized that knocking down *DPYSL3* in UC cells suppressed mTOR signalling. Immunoblot analysis revealed that KD clones had decreased the protein levels of mTOR, phospho-RPS6 (p-RPS6(S235)), and MYC** (**Fig. 6a**).** The expression of AKT, phospho-AKT (p-AKT(S473)), and phospho-mTOR (p-MTOR(S2448)) was decreased in shDPYSL3#1 T24 cells but not in shDPYSL3#1 BFTC909. Although the active/phospho-mTOR is not consistently suppressed, the downregulation of phospho-RPS6 upon *DPYSL3* knockdown indicated that the mTORC1 activity was affected. These findings suggested that DPYSL3 can enhance mTORC1 signalling in UC cells, which would be predicted to promote aerobic glycolysis. In addition, DPYSL3 confers UC aggressiveness is associated with activating the mTOR pathways and cMYC overexpression.

### DPYSL3 knockdown UC cells downregulated GLUT1 expression and hampered tumour growth in vivo

The mTOR pathway is a vital regulator in cancer cells to induce aerobic glycolysis, a crucial feature in UBUC [33, 34]. Immunoblot analysis revealed that DPYSL3 knockdown in BFTC909 and T24 significantly suppressed GLUT1 expression but did not affect HK2 and LDHA/C expression (Fig. 6a). These findings indicate that DPYSL3 stimulated glucose influx through increased GLUT1 expression, fuelling the mTOR signal.

The mouse xenograft experiments showed that the growth of tumours derived from BFTC909 cells was hampered after DPYSL3 knockdown. The average tumour size was significantly smaller in the BFTC909 sh*DPYSL3* group than in the BFTC909 sh*LacZ* group (*p* < 0.05, Figs. 6b and c). The tumours developed from BFTC909 sh*DPYSL3* cells had lower cMYC and GLUT1 expression (Fig. 6d). Based on these findings, we revealed that DPSYL3 could promote rapid UC growth by cMYC overexpression and increased glucose influx in UC. The link between DPYSL3 and cancer energy metabolism has never been reported and thus warrants further investigation.

## Discussion

Rapid growth and sustained proliferation are hallmarks of malignant tumours [35]. To maintain expedited cell cycle progression, a continually replenished nucleotide pool is required to support DNA and RNA synthesis. The metabolic shift to a higher de novo nucleotide synthesis rate is thus crucial to supporting tumour development and progression [36]. Upon data mining of the GEO dataset GSE31684, we found that DPYSL3 expression was significantly correlated with UBUC patient prognosis in a manner focused on the nucleobase-containing compound metabolic process (GO:0006139). Analyses with our well-documented cohorts confirmed that increased DPYSL3 protein expression in UTUC and UBUC is associated with poor DFS and MFS. In addition, high DPYSL3 expression predicted a higher bladder tumour recurrence rate in patients with NMIBC. This finding suggests that DPYSL3 positively affects tumour aggressiveness and plays an essential role in the tumorigenesis of UC.

Activation of invasion and metastasis is one of the hallmark capabilities of cancer cells. Cancer cells have two distinct modes of invasion: “collective invasion” and “single-cell migration” [35]. The former is characterized by the invasive growth of whole groups of tumour cells into adjacent tissue interconnected by adhesion molecules and communication junctions [37, 38]. The latter is characterized by the invasion of individual tumour cells into the surrounding stroma [38]. Dynamic modification of the actin cytoskeleton is required for cell migration, playing a vital role in the invasion and metastasis of cancer cells [39]. In claudin-low breast cancer cells, DPYSL3 mRNA and the epithelial-mesenchymal transition (EMT) markers SNAIL and TWIST can regulate each other reciprocally [40]. In addition, DPSYL3-silenced lung cancer cells overexpress epithelial markers and downregulated mesenchymal markers [24]. Since our in vitro and in vivo results revealed that DPYSL3 increased UC aggressiveness by promoting tumour cell proliferation, migration, and invasion, it is possible that DPYSL3 confers aggressive behaviours on UC cells through profound modulation of the UC cell actin cytoskeleton, boosts epithelial-mesenchymal transition, and promotes cell mitosis.

In addition to modifying UC cell invasiveness, DPYSL3 is involved in diverse molecular processes to promote tumour aggressiveness. Our experiment showed that DPYSL3 knockdown in UC not only promotes G1-phase arrest but also enhances UC cell apoptosis. A previous study in gastric cancer showed that modulating the expression of the short DPYSL3 isoform induces G1-phase arrest but not apoptosis [41]. In addition, decreased DPYSL3 expression in UC cells suppressed tumour angiogenesis. Our previous study has shown that an increased microvascular density in UC tumours is associated with poor patient survival outcomes [42]. DPYSL3 could be used as a biomarker to identify patients most likely to benefit from antiangiogenic therapeutics. The mechanism of DPYSL3-promoted angiogenesis in UC is still unknown, but a previous study showed that the expression of DPYSL3 and VEGF was correlated [20].

Metabolic reprogramming (the Warburg effect) is critical for cancer cell proliferation. In addition to rapid ATP synthesis, metabolic process remodelling supports the biosynthetic pathways and promotes signalling transduction crucial for cancer cells [43]. Alterations in the metabolic pathway can promote tumorigenesis and EMT [44, 45]. From the Gene Ontology analysis, we discovered that genes associated with DPYSL3 were also enriched in functions related to metabolic processes. We revealed that DPYSL3 overexpression correlates with increased MYC and GLUT-1 expression using a mouse xenograft model. We also demonstrated that DPYSL3 expression correlates with mTOR pathway activation in UC cell lines. mTOR induces metabolic reprogramming in tumours by boosting protein and lipid synthesis and aerobic glycolysis to fuel tumour cells [46]. Based on the findings, we suggested that DPYSL3 induced GLUT1 expression in UC cells, which led to glucose influx into the cells. Increased intracellular glucose can activate mTORC1 by increasing Rheb interaction [47]. With the upregulated cMYC induced by DPYSL3, mTORC1 reprogrammed UC metabolism by promoting glutaminolysis, glycolysis, and nucleotide synthesis [48].

This study had two significant limitations that must address in future studies. First, this is a single institute study, and the tissue samples collected are from a region where UTUC has a high prevalence. The etiology of UC development may be, and therefore, our findings might not apply to different populations. A multicenter, multi-continental study will be needed to confirm our observations. Second, our result only demonstrated that UC with DPYSL3 knockdown is associated with decreased MYC and GLUT1 expression; how DPYSL3 promotes energy reprogramming has yet to clarify. Reduced phosphorylation of mTOR and RPS6 may be essential for DPYSL3 in altering the energy metabolism of UC. A comprehensive study on DPYSL3 interaction with tumour cell energy metabolism will be needed to understand the underlying molecular mechanism. Despite this limitation, our study is the first to disclose DPYSL3 in reprogramming energy metabolism in cancer development**.**

## Conclusions

DPYSL3 is an important biomarker that correlates with UC aggressiveness and independently predicts UTUC and UBUC outcomes. The oncogenic roles of DPYSL3 in UC involved cytoskeleton modification, promoting cell proliferation, preventing apoptosis, induced angiogenesis, and reprogramming metabolic processes. DPYSL3 confers tumour aggressiveness by increased cMYC and GLUT1 expression and activation of mTORC1 signalling. DPYSL3 could serve as a prognostic marker and a potential therapeutic target for UC.

## Supplementary Information

**Additional file 1: Table S1.** Primary antibodies and dilution for immunohistochemistry and immunoblot analysis.

**Additional file 2: Table S2.** The flow cytometry assay of the Cell Cycle of mock and DPYSL3 knockdown UC cells (BFTC909 and T24).

**Additional file 3: Figs. S1-S7.** Immunoblot Gel Raw data.

**Additional file 4: Table S3.** The top 500 most significant differentially expressed genes positively correlated with *DPYSL3* expression in bladder cancer.

**Additional file 5: Table S4.** The top 500 most significant differentially expressed genes negatively correlated with *DPYSL3* expression in bladder cancer.

**Additional file 6: Table S5.** GO enrichments of positively associated genes.

**Additional file 7: Table S6.** GO enrichments of negatively associated genes.

## Data Availability

The datasets generated and analyzed during the current study are not publicly available since the storage of the data in a public register is not covered by the patient’s declaration of consent. However, an anonymous data set is available from the corresponding author [C.-F. L] on reasonable request.

## Abbreviations

CLOW: Claudin-low
CRMP4: Collapsing response mediator protein 4
DPYSL3: Dihydropyrimidinase-like 3 protein
DSS: Disease-specific survival
EMT: Epithelial-mesenchymal transition
FFPE: Formalin-fixed paraffin-embedded
GEO: Gene Expression Omnibus
GEPIA: Gene Expression Profiling Interactive Analysis
GO: Gene Ontology
GSK3b: Glycogen synthase kinase 3 beta
HUVECs: Human umbilical vein endothelial cells
LRFS: Local recurrence-free survival
MFS: Metastatic free survival
MIBC: Muscle-invasive bladder cancer
NMIBC: Non-muscle invasive bladder cancer
pT: Primary tumour stage
TCGA: The Cancer Genome Atlas
TMA: Tissue microarrays
UBUC: Urinary bladder urothelial carcinoma
UC: Urothelial carcinoma
UTUC: Upper urinary tract urothelial carcinoma
VEGF: Vascular epithelial growth factor
