# Supplementary material for: Upregulation of dihydropyrimidinase-like 3 (DPYSL3) protein predicts poor prognosis in urothelial carcinoma
Source: BMC Cancer. 2023 Jun 28;23:599. doi: 10.1186/s12885-023-11090-z (PMC10304234; doi:10.1186/s12885-023-11090-z)
Supplement: Supplementary file 1 — Additional file 1: Table S1. Primary antibodies and dilution for immunohistochemistry and immunoblot analysis. [file 12885_2023_11090_MOESM1_ESM.pdf]

Table S1.

a. Primary antibodies and dilution for immunohistochemistry

Symbol/antibody	Protein	Dilution/Cat./Company
DPYSL3	Dhydropyrimidinase-like 3	1:100, GTX16407, GeneTex Ltd
GLUT1	Glucose transporter 1	1:200, #12939, Cell Signaling
MYC	Myelocytomatosis	1:1000, ab32072, Abcam

b. Primary antibodies and dilution for immunoblot analysis

Symbol/antibody	Protein	Dilution/Cat./Company
DPYSL3	Dhydropyrimidinase-like 3	1:10000, GTX16407, GeneTex Ltd
GLUT1	Glucose transporter 1	1:1000, #12939, Cell Signaling
HK2	Hexokinase 2	1:1000, #2867, Cell Signaling
LDHA/C	Lactate dehydrogenase A/C	1:1000, #3558, Cell Signaling
MTOR	Mechanistic target of rapamycin kinase	1:500, ab32028, Abcam
AKT	AKT serine/threonine kinase	1:2000, #4691, Cell Signaling
p-AKT(S473)	Phospho-AKT1 at serine 473	1:2000, #4060, Cell Signaling
p-MTOR(S2448)	Phospho-MTOR at serine 2448	1:500, ab51044, Abcam
p-RPS6(S235)	Phospho-RPS6 at serine 235	1:20000, ab80158, Abcam
RPS6	Ribosomal protein S6	1:1000, ab137826, Abcam
