# Supplementary material for: Upregulation of dihydropyrimidinase-like 3 (DPYSL3) protein predicts poor prognosis in urothelial carcinoma
Source: BMC Cancer. 2023 Jun 28;23:599. doi: 10.1186/s12885-023-11090-z (PMC10304234; doi:10.1186/s12885-023-11090-z)
Supplement: Supplementary file 2 — Additional file 2: Table S2. The flow cytometry assay of the Cell Cycle of mock and DPYSL3 knockdown UC cells (BFTC909 and T24). [file 12885_2023_11090_MOESM2_ESM.pdf]

Table S2. The Flow Cytometry Assay of Cell Cycle of Mock and DPYSL3 Knockdown UC Cells (BFTC909 and T24)

BFTC909	shLacZ		shDPYSL3#1			shDPYSL3#2		
	Mean	SD	Mean	SD	Sig.	Mean	SD	Sig.
SubG1 Phase	0.19	0.08	2.14	0.09	P <0.05	2.08	0.09	P <0.05
G0/G1 Phase	29.3	1.65	43.6	1.81	P <0.05	45.7	1.79	P <0.05
S Phase	42.6	1.75	30.8	1.67	P <0.05	29.7	1.05	P <0.05
G2M Phase	29.9	1.98	17.5	1.21	P <0.05	16.9	1.77	P <0.05

T24	shLacZ		shDPYSL3#1			shDPYSL3#2		
	Mean	SD	Mean	SD	Sig.	Mean	SD	Sig.
SubG1 Phase	0.11		0.16		n.s	0.15		n.s
G0/G1 Phase	65.6	0.87	72.1	2.98	P <0.05	76.2	2.61	P <0.05
S Phase	18.6	1.65	16.4	0.27	P <0.05	15.3	0.2	P <0.05
G2M Phase	12.7	1.16	9.2	0.35	P <0.05	8.1	0.44	P <0.05
