# Supplementary material for: Upregulation of dihydropyrimidinase-like 3 (DPYSL3) protein predicts poor prognosis in urothelial carcinoma
Source: BMC Cancer. 2023 Jun 28;23:599. doi: 10.1186/s12885-023-11090-z (PMC10304234; doi:10.1186/s12885-023-11090-z)
Supplement: Supplementary file 3 — Additional file 3: Figs. S1-S7. Immunoblot Gel Raw data. [file 12885_2023_11090_MOESM3_ESM.pdf]

DPYSL3 Expression in UC Cell Lines

DPYSL3 (ab126787)
1:10000   62 kDa

Data sheet

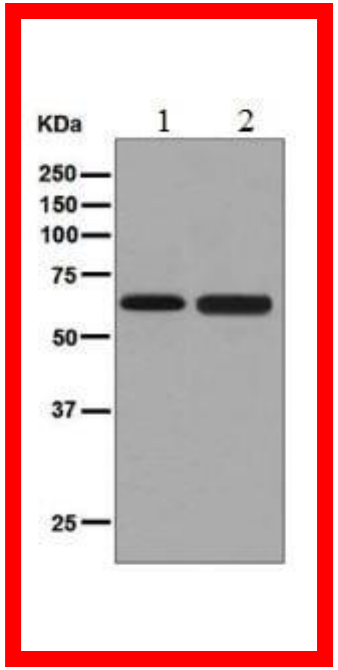

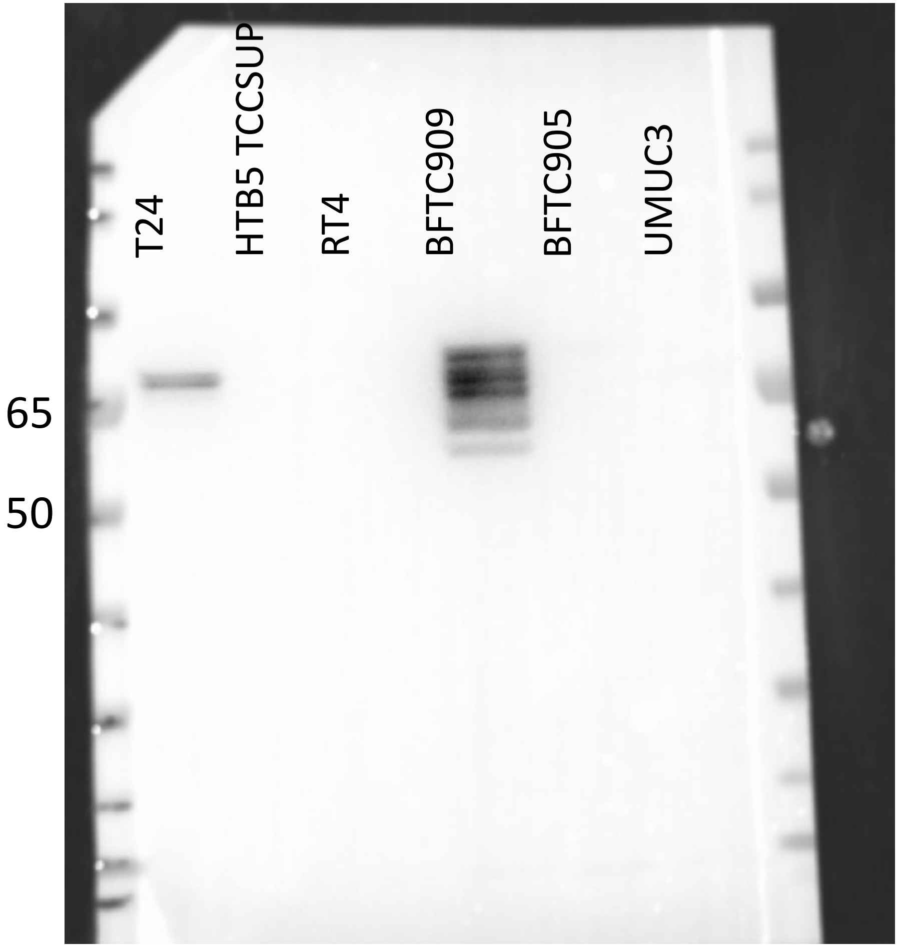

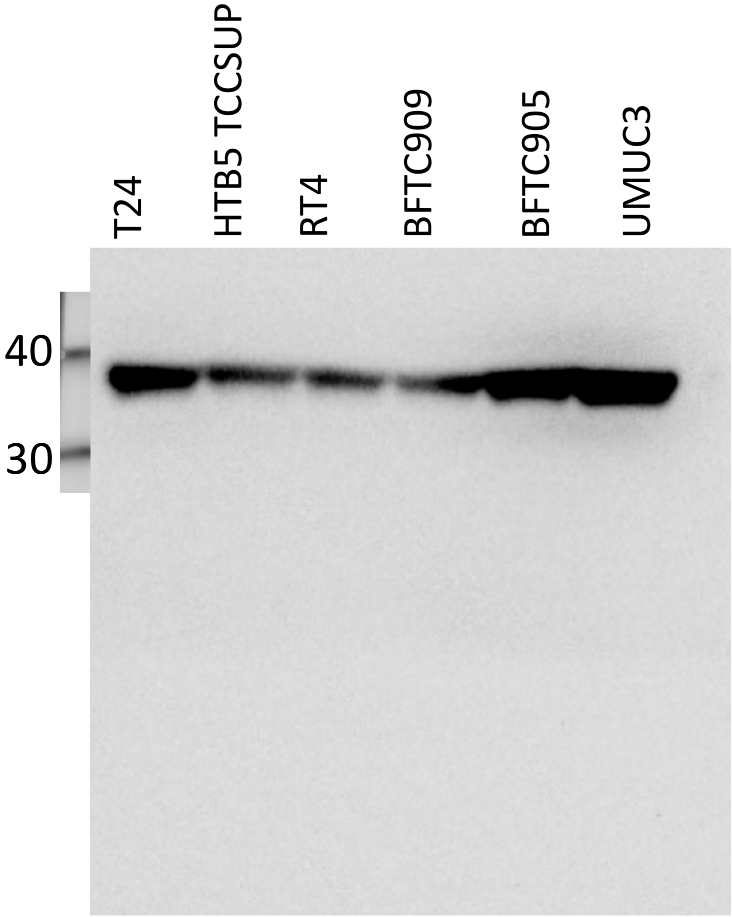

GAPDH (ab181602)
1:10000   37 kDa

Validation of shDPYSL3 KD in T24

Data sheet

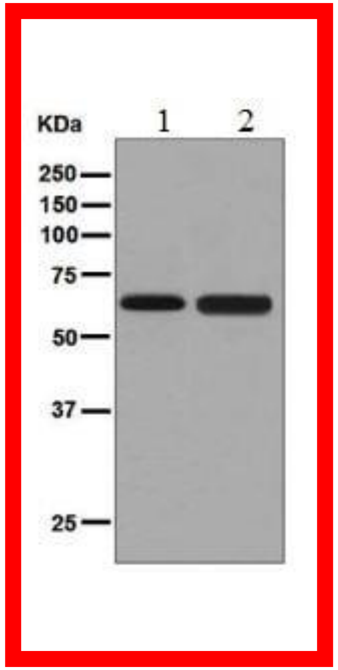

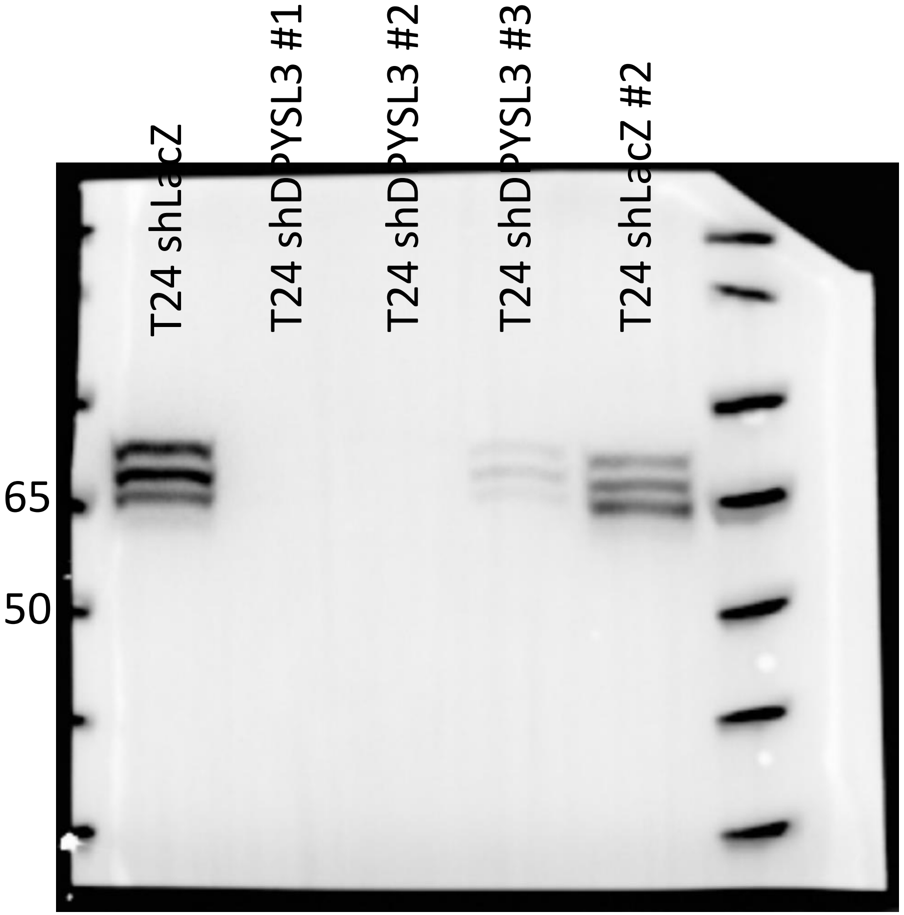

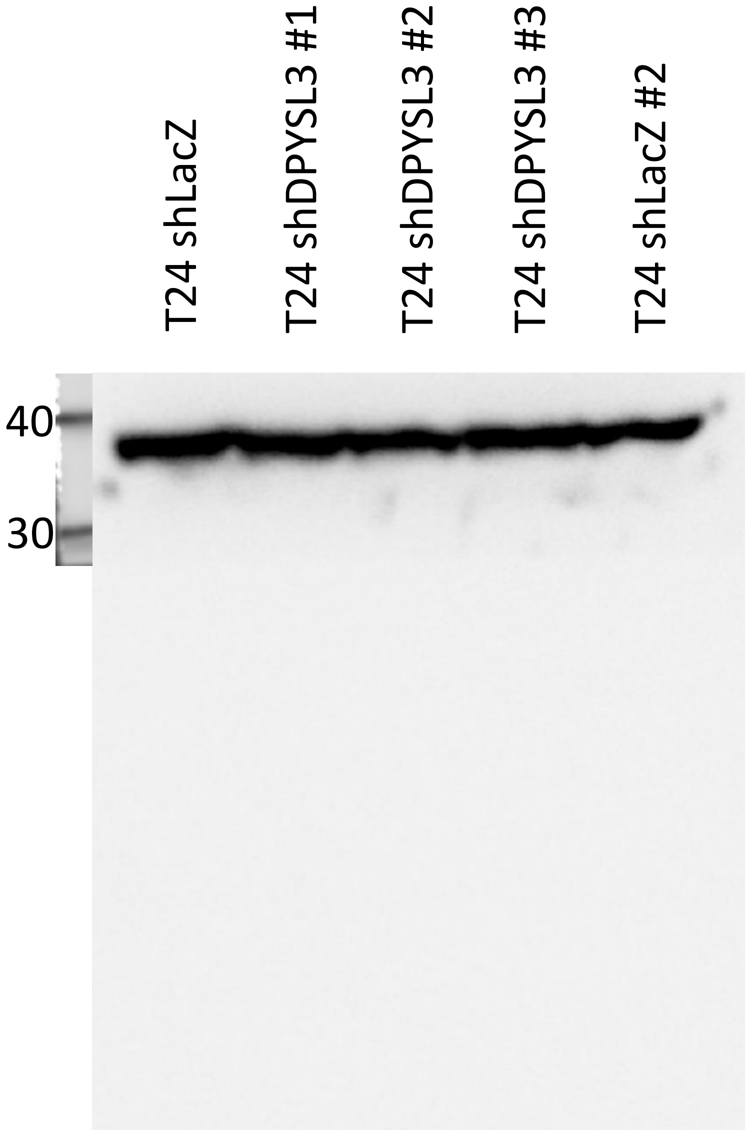

GAPDH (ab181602)
1:10000 37 kDa

Validation of shDPYSL3 KD in BFTC909

DPYSL3 (ab126787)
1:10000   62 kDa

Data sheet

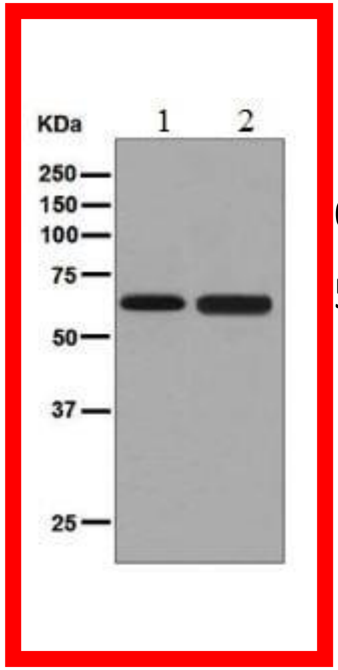

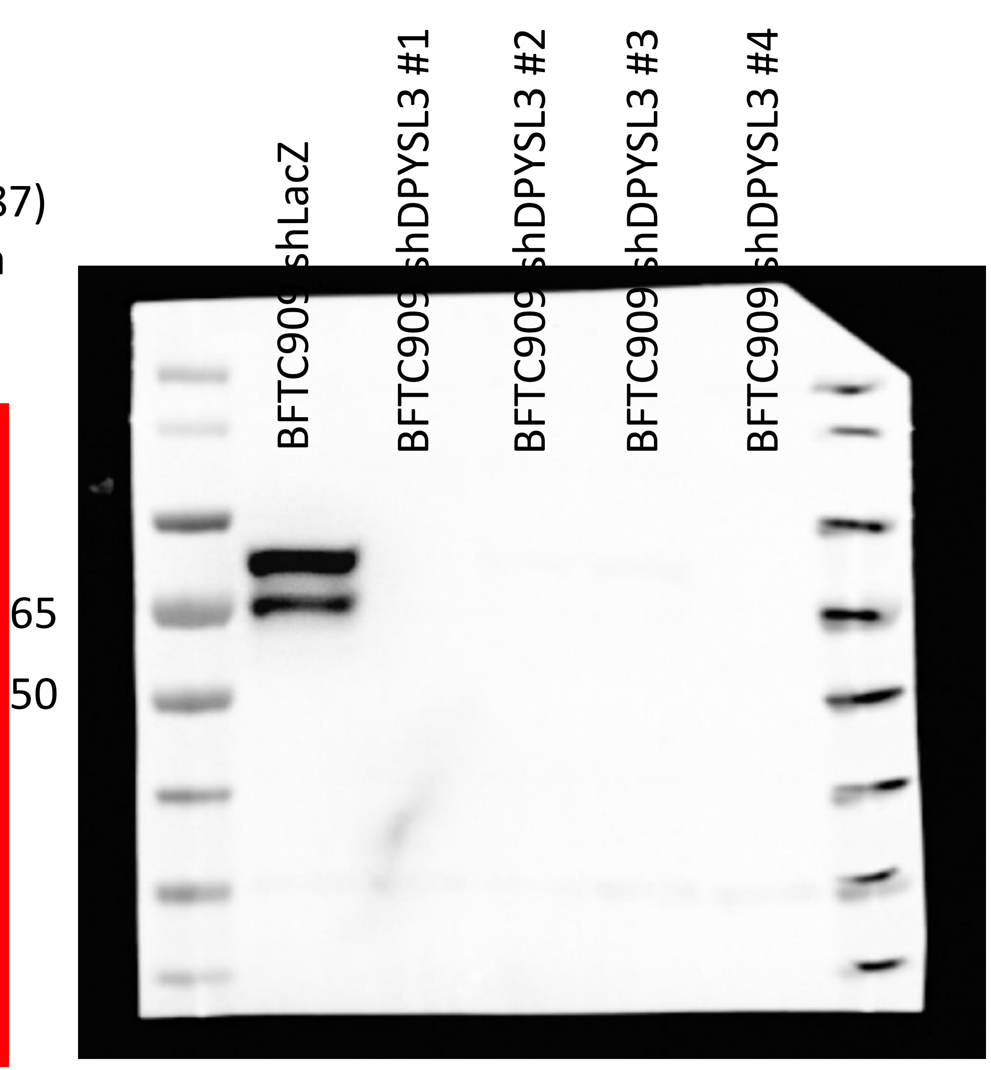

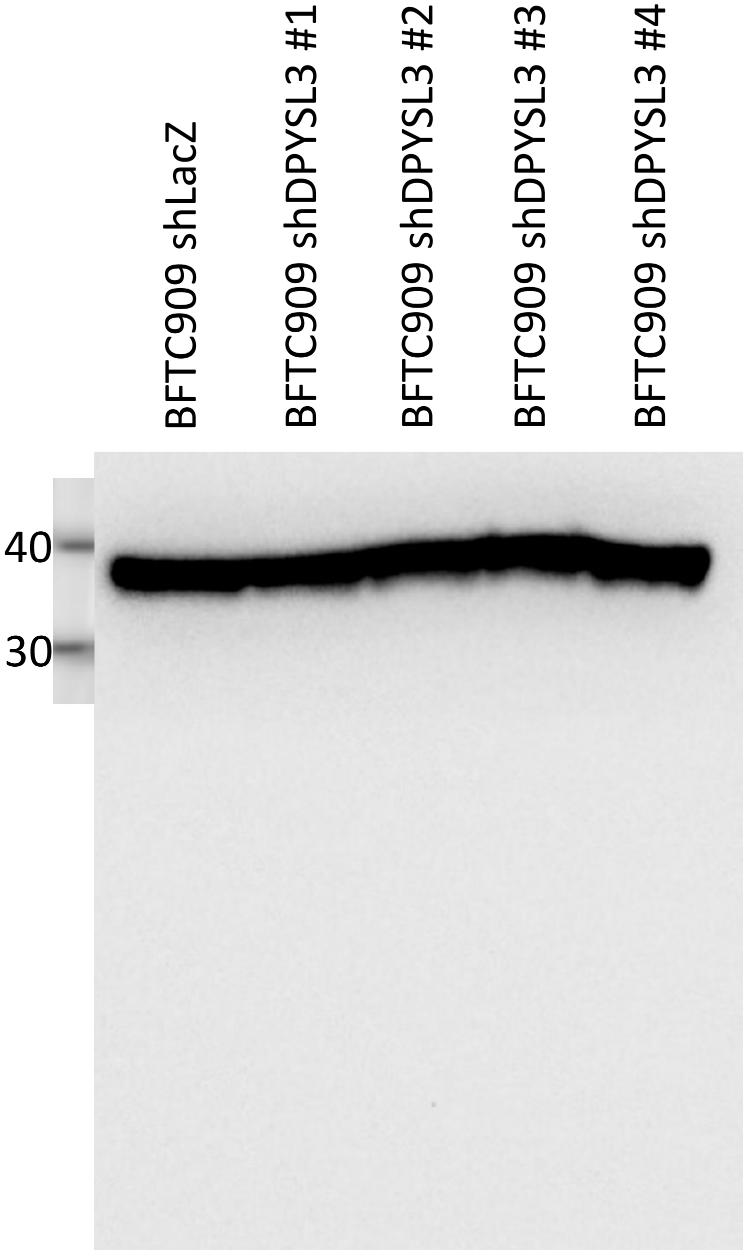

GAPDH (ab181602)
1:10000   37 kDa

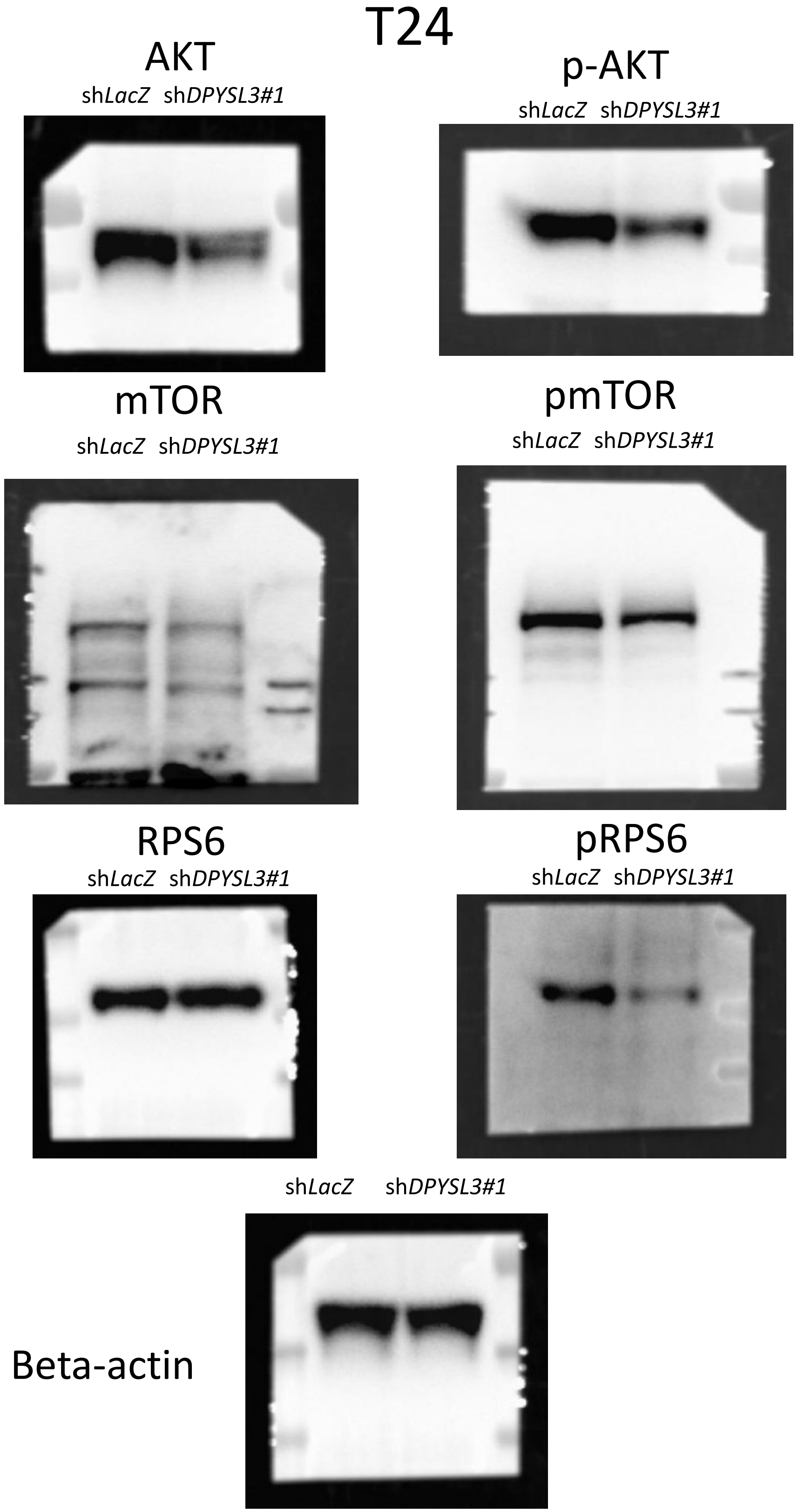

T24
AKT
shLacZ shDPYSL3#1
p-AKT
shLacZ shDPYSL3#1
mTOR
shLacZ shDPYSL3#1
pmTOR
shLacZ shDPYSL3#1
RPS6
shLacZ shDPYSL3#1
pRPS6
shLacZ shDPYSL3#1
shLacZ shDPYSL3#1
Beta-actin

T24

MYC

sh*LacZ* sh*DPYSL3#1*

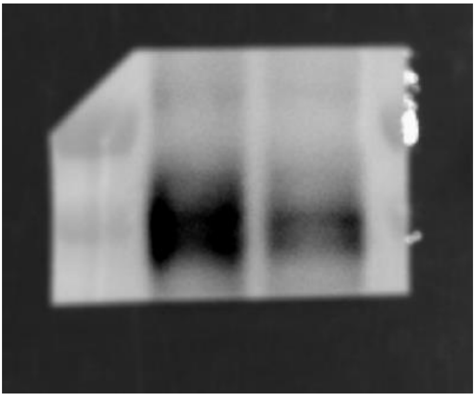

GLUT1

sh*LacZ* sh*DPYSL3#1*

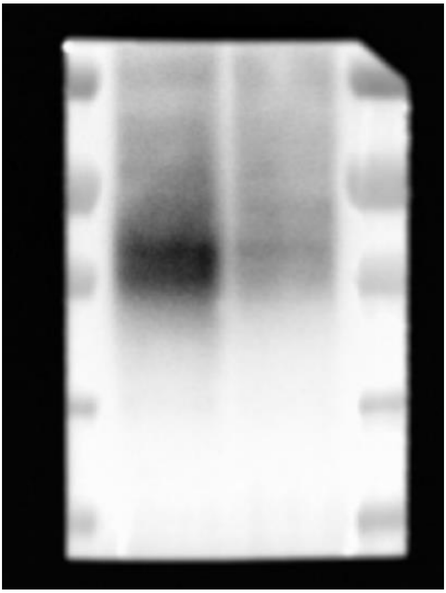

HK2

sh*LacZ* sh*DPYSL3#1*

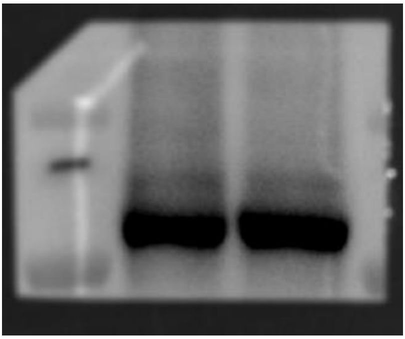

LDHAC

sh*LacZ* sh*DPYSL3#1*

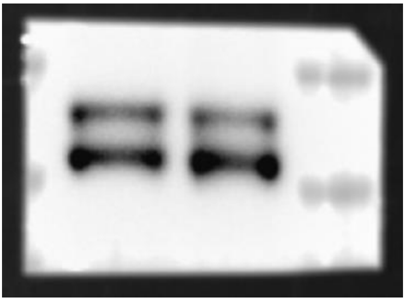

sh*LacZ* sh*DPYSL3#1*

B-actin

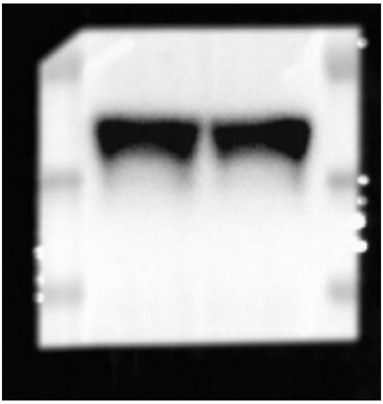

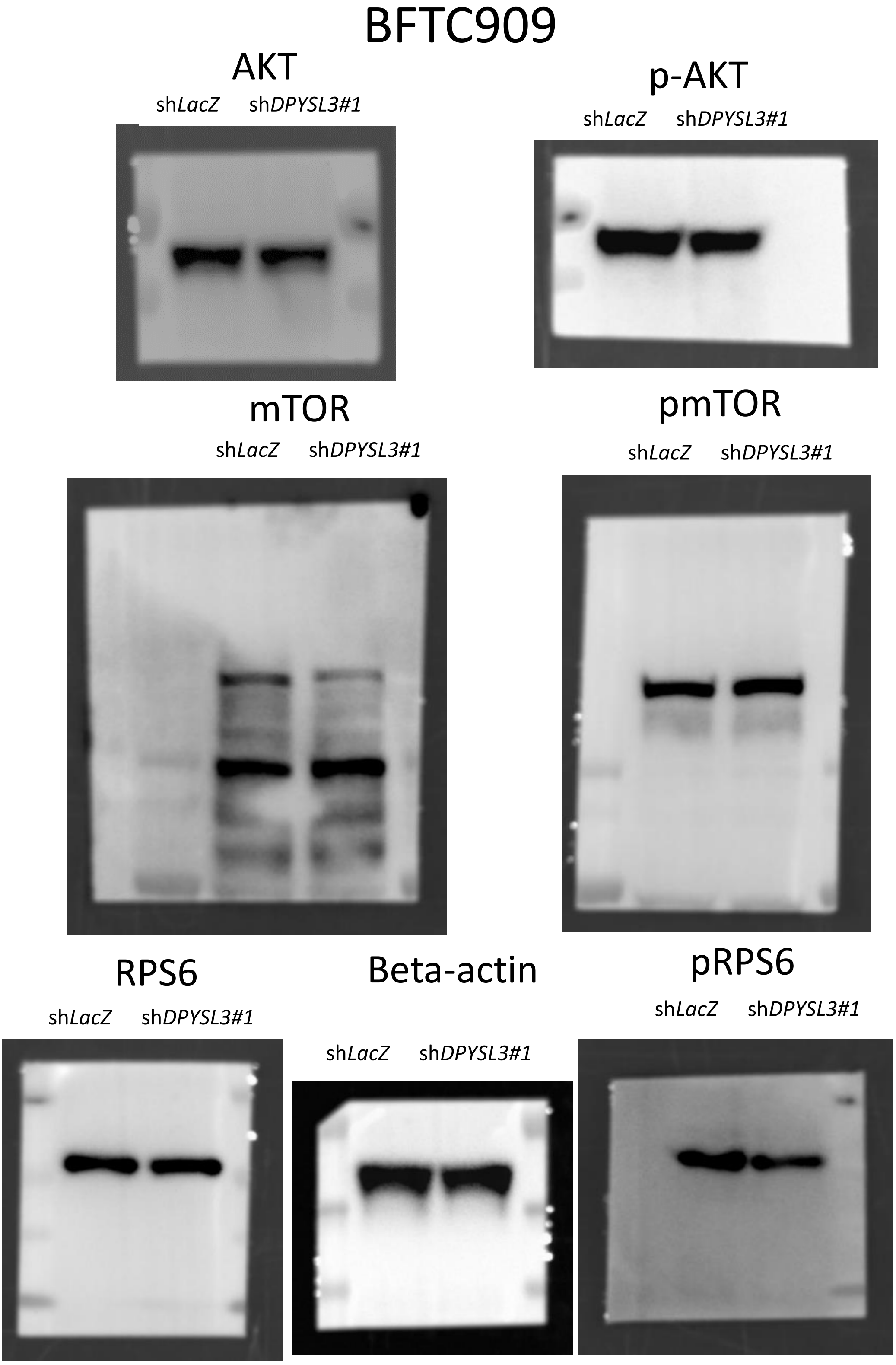
BFTC909
AKT
shLacZ shDPYSL3#1
p-AKT
shLacZ shDPYSL3#1
mTOR
shLacZ shDPYSL3#1
pmTOR
shLacZ shDPYSL3#1
RPS6
shLacZ shDPYSL3#1
Beta-actin
shLacZ shDPYSL3#1
pRPS6
shLacZ shDPYSL3#1

BFTC909

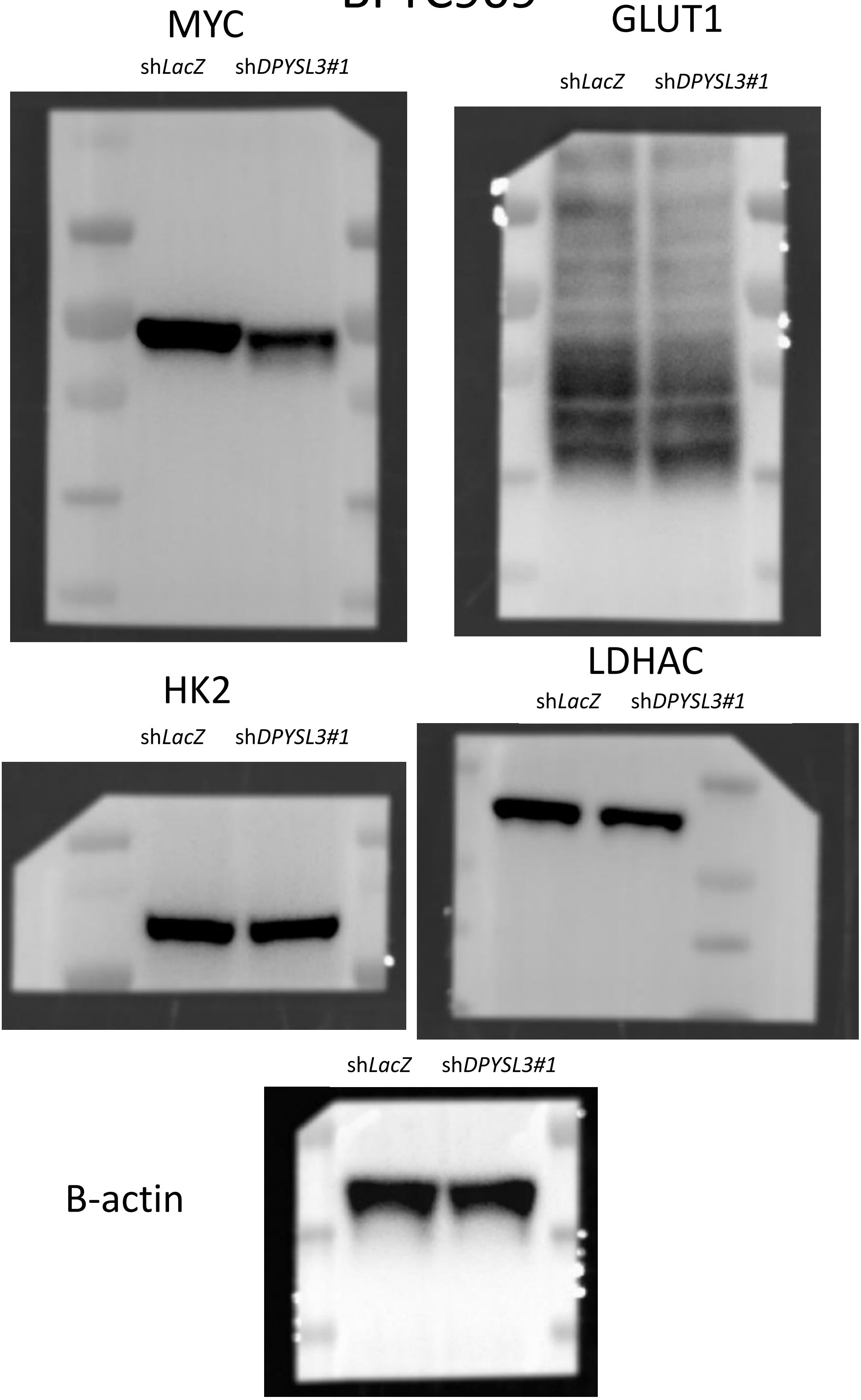
MYC
shLacZ shDPYSL3#1
GLUT1
shLacZ shDPYSL3#1
HK2
shLacZ shDPYSL3#1
LDHAC
shLacZ shDPYSL3#1
shLacZ shDPYSL3#1
B-actin
